# Acetylcholinesterase- and Butyrylcholinesterase-Based Biosensors for the Detection of Quaternary Ammonium Biocides in Food Industry

**DOI:** 10.3390/foods13010133

**Published:** 2023-12-30

**Authors:** Lynn Mouawad, Georges Istamboulie, Gaëlle Catanante, Thierry Noguer

**Affiliations:** 1 Biosensors Analysis Environment Group (BAE-LBBM), Université de Perpignan, Via Domitia, 52 Avenue Paul Alduy, Cedex, F-66860 Perpignan, France; lynn.mouawad@univ-perp.fr (L.M.); georges.istamboulie@univ-perp.fr (G.I.); gaelle.catanante@univ-perp.fr (G.C.); 2 Laboratoire de Biodiversité et Biotechnologie Microbienne (LBBM), Sorbonne Université, CNRS, UAR 3579, Observatoire Océanologique, F-66650 Banyuls-sur-Mer, France

**Keywords:** quaternary ammoniums, biocides, cholinesterases, biosensor, screen-printed electrodes, phosphotriesterase

## Abstract

A sensitive and robust electrochemical cholinesterase-based sensor was developed to detect the quaternary ammonium (QAs) biocides most frequently found in agri-food industry wash waters: benzalkonium chloride (BAC) and didecyldimethylammonium chloride (DDAC). To reach the maximum residue limit of 28 nM imposed by the European Union (EU), two types of cholinesterases were tested, acetylcholinesterase (AChE, from *Drosophila melanogaster*) and butyrylcholinesterase (BChE, from horse serum). The sensors were designed by entrapping AChE or BChE on cobalt phthalocyanine-modified screen-printed carbon electrodes. The limits of detection (LOD) of the resulting biosensors were 38 nM for DDAC and 320 nM for BAC, using, respectively, AChE and BChE. A simple solid-phase extraction step was used to concentrate the samples before biosensor analysis, allowing for the accurate determination of DDAC and BAC in tap water with limits of quantification (LOQ) as low as 2.7 nM and 5.3 nM, respectively. Additional assays demonstrated that the use of a phosphotriesterase enzyme allows for the total removal of interferences due to the possible presence of organophosphate insecticides in the sample. The developed biosensors were shown to be stable during 3 months storage at 4 °C.

## 1. Introduction

**Figure 1:**
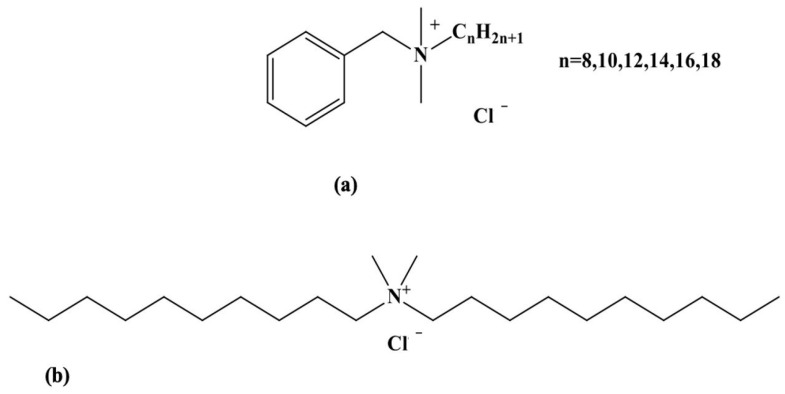
Chemical structures of BAC (**a**) and DDAC (**b**) biocides.

Disinfectant biocides are chemicals heavily used by the food industry to control the microbiological contamination of surfaces in contact with food products intended for human consumption [1]. They were initially employed for food and water conservation, although there are early reports of their use in wound cleansing [2]. Biocides are also widely used in healthcare environments, particularly for disinfecting and sterilizing surfaces and medical equipment [3]. Different chemicals have been developed over the years, including phenols, chlorine-releasing agents (CRAs), alcohols, iodine, hydrogen peroxide, and silver compounds. In the 20th century, other chemical agents such as quaternary ammonium (QA) compounds were introduced and used for various applications at suitable concentrations [4]. Owing to their low cost and broad biocidal spectrum towards bacteria, fungi, parasites, and viruses, benzalkonium chloride (BAC) and didecyldimethylammonium chloride (DDAC) are the most used QA-based biocides in the agri-food industry, and more specifically for the disinfection of dairy industry equipment [1,5] (Figure 1). QA compounds are effective in eliminating microorganisms, but various studies have indicated that these surface agents adsorb onto the equipment and resist washing, and that some of their residues are transferred to the food, causing many health issues ranging from gastrointestinal problems to coma and death [6]. A study conducted by the European Food Safety Authority (EFSA) has demonstrated the presence of BAC and DDAC residues in 12% of milk samples tested, 6% in leafy vegetables, and 5% in infant food [7]. Moreover, biocides are not biodegradable, posing a potential risk to the environment [8]. Although data on the detection of these biocides in the environment are scarce in the literature, BAC concentrations have been reported in milligrams per liter in hospital wastewater. In addition, wastewater plants are unable to treat QA contaminants, resulting in their release as micropollutants into the environment [9]. For that reason, the regulatory framework EU regulation 1119/2014 for the presence of biocides in specific foods such as milk established a maximum residue limit for BAC and DDAC of 0.1 mg/Kg. A default standard biocide limit of 0.01 mg/kg was also established for wash water, equivalent to 28 nM [10].

Several analytical techniques have been reported in the literature for the detection of biocides. Liquid chromatography combined with mass spectroscopy (LC-MS-MS) was used to detect BAC and DDAC with a very low LOD of 0.1 µg/L [11]. However, these techniques present some disadvantages, as they cannot be used for on-site detection, they require trained personnel and sophisticated equipment requiring costly maintenance [12]. As EU policies impose routine testing on the agri-food industry for safety purposes, it appears necessary to develop a sensitive, cost-effective, and easy-to-use device for detecting these biocides in food and wastewater in real time. A study published in 1952 reported that acetylcholinesterase (AChE) from electric eel (*Electrophorus electricus*) was reversibly inhibited by certain quaternary ammonium salts [13]. This discovery opens the possibility of developing a cholinesterase (ChE) biosensor for the detection of these biocides based on the enzyme inhibition process. Cholinesterase electrochemical biosensors were developed in the early 1960. The first device based on ChE inhibition by some organophosphorus compounds was described in 1962. Since then, multiple ChE biosensors have been developed for the detection of different toxic substances such as nerve agents and pesticides, which act as irreversible inhibitors. The source of the selected enzyme greatly affects the biosensor’s performance and sensitivity. For instance, AChE extracted from insects are used preferentially for the detection of insecticides due to their higher sensitivities towards their targets. In addition, some studies have focused on the use of recombinant ChE enzymes to further decrease the sensitivity of biosensor devices [14].

Although ChE-based biosensors have demonstrated their great potential due to their relative low cost, simple use and sensitivity towards insecticides, similar sensors described in literature did not show sufficient sensitivity for detecting quaternary ammonium biocides [15].

The aim of the present study was therefore to develop a novel cholinesterase biosensor capable of detecting BAC and DDAC biocides in tap water at low concentrations based on cholinesterase inhibition. Preliminary tests were conducted by testing two types of cholinesterases, acetylcholinesterase and butyrylcholinesterase (BChE), to determine the most sensitive enzyme against the selected biocides. In a second step, amperometric enzyme biosensors were developed based on entrapment of the most suitable enzyme on the surface of cobalt phthalocyanine-modified screen-printed carbon electrodes. In addition, a test using phosphotriesterase enzyme (PTE) was developed in order to eliminate interfering substances such as organophosphate compounds (OPs), which are irreversible inhibitors of cholinesterases. PTE enzyme, also known as organophosphate hydrolase (OPH), has the ability of hydrolyzing organophosphates [16]. This enzyme, when added to the reaction medium, allowed us to discriminate efficiently between biocides and OPs.

To the best of our knowledge, the described biosensor is the first device capable of detecting BAC and DDAC biocides in a convenient concentration range while discriminating between biocides and OPs.

## 2. Materials and Methods

### 2.1. Materials

AChE from *Drosophila melanogaster* (wild type) was produced by the Centre de Recherche de Biochimie Macromoléculaire (CRBM) (Montpellier, France), while horse serum BChE was obtained from Sigma-Aldrich (St. Louis, MO, USA). Acetylthiocholine chloride and iodide were purchased from Sigma-Aldrich (St. Louis, MO, USA), and butyrylthiocholine iodide was obtained from Thermo Fisher Scientific (Kandle, Germany). The activity of cholinesterases was measured optically in the presence of 5,5′-dithiobis (2-nitrobenzoic acid) (DTNB-Ellman’s reagent), purchased from Sigma-Aldrich. BAC (C12-C18) and DDAC quaternary ammoniums were purchased from Sigma-Aldrich. Phosphotriesterase (PTE) was produced by Protein Bio Sensor (Toulouse, France). Paraoxon used as a PTE substrate was obtained from Riedel-de Häen (Seelze, Germany). All solutions were prepared daily in deionized water prior to each measurement. Biosurfine-MRH photopolymer (PVA) was used for enzyme immobilization and was kindly provided by Toyo Gosei Kogyo Co. (Chiba, Japan). Poly (vinyl) chloride (PVC) sheets (200 mm × 100 mm x 0.5 mm) (SKK, Denzlingen, Germany) were used as support for the screen-printed electrodes. Graphite (Electrodag 423SS) and silver/silver chloride (Electrodag 6037SS) screen-printing pastes were obtained from Acheson (Plymouth, UK). Cobalt phthalocyanine (Co-PC)-modified carbon paste was purchased from Gwent Electronic Materials, Ltd. (Gwent, UK). A glycerophthalic paint (AkzoNobel, Amsterdam, Netherlands) was used as insulating layer. Oasis HLB solid-phase extraction cartridges used for samples pretreatment were purchased from Waters (Milford, MA, USA). 0.1 M PBS buffers at pH 7 and pH 8 used in all experiments were made of sodium phosphate dibasic (Na_2_HPO_4_) and potassium dihydrogen phosphate (KH_2_PO_4_) supplemented with 0.1 M potassium chloride (KCl).

### 2.2. Methods

#### 2.2.1. Determination of Enzymes Activity

Cholinesterase (ChEs) activity was measured in PBS at pH 7 by spectrophotometry, using a Shimadzu UV-1800 spectrophotometer. In the presence of the enzyme, the substrate acetylthiocholine (AtCh) or butyrylthiocholine (BtCh) (depending on the cholinesterase type) is hydrolyzed into thiocholine, which in turn reacts with Ellman’s reagent 5,5′-dithiobis-2-nitrobenzoic acid (DTNB), leading to the formation of 5-thio-2-nitrobenzoic acid (TNB), a yellow product measured at 412 nm (ε = 14,150 M^−1^ cm^−1^) [17,18]. 1 enzyme unit (U) was defined as the amount of enzyme allowing for the transformation of 1 µmol of substrate per minute. ChEs solutions at 0.33 U/mL were prepared and stored at 4 °C before use.

Phosphotriesterase (PTE) activity was determined in PBS at pH 8 by spectrophotometry. In the presence of the enzyme, the substrate paraoxon is hydrolyzed into paranitrophenol (PNP), which can be measured at 405 nm (ε = 16,800 M^−1^ cm^−1^) [19]. It is important to stress that this product is not an inhibitor of cholinesterases and is not toxic nor classified as harmful by the WHO and the European regulation on water quality [20]. PTE solutions at 0.8 U/mL were prepared and stored at 4 °C before use.

#### 2.2.2. Biosensor Implementation

The screen-printed electrodes used for the electrochemical measurements were fabricated in our laboratory using a DEK248 printing machine according to a previously described method [21]. The three-electrode system consisted of a Co-PC-modified carbon working electrode (4 mm diameter), a straight Ag/AgCl reference electrode (5 mm × 1.5 mm), and a curved carbon counter electrode (16 mm × 1.5 mm). Electrodes were cured at 60 °C during 3 h after each layer deposition. A cobalt phthalocyanine mediator was integrated in the carbon paste. This step allowed for a reduction in the applied potential and electrochemical interferences, improving the stability and the reproducibility of the biosensor.

Enzyme immobilization by entrapment methods is well known to enhance the stability of enzymes by decreasing their denaturation, leading to an improvement in enzymatic biosensor lifespan [22]. In this work cholinesterase enzymes (AChE or BChE) were immobilized on the surface of the working electrode by entrapment in a polyvinyl alcohol photosensitive polymer (Biosurfine-MRH). An enzyme solution in PBS buffer at pH 7 containing 0.33 U/mL of AChE or BChE was mixed with the polymer in a 70%:30% ratio (*v/v*) [18]. 3 µL of the resulting mixture was spread on the surface of the working electrode using a micropipette. The resulting amount of enzyme immobilized on the working electrode was calculated to be 0.3 mU. The modified electrode was placed under 2 white neon lights (Philips T5 short, 4000 K, 8 W, 380 lm) for 48 h to allow for the photopolymerization process and then was stored at 4 °C before use.

**Figure 2:**
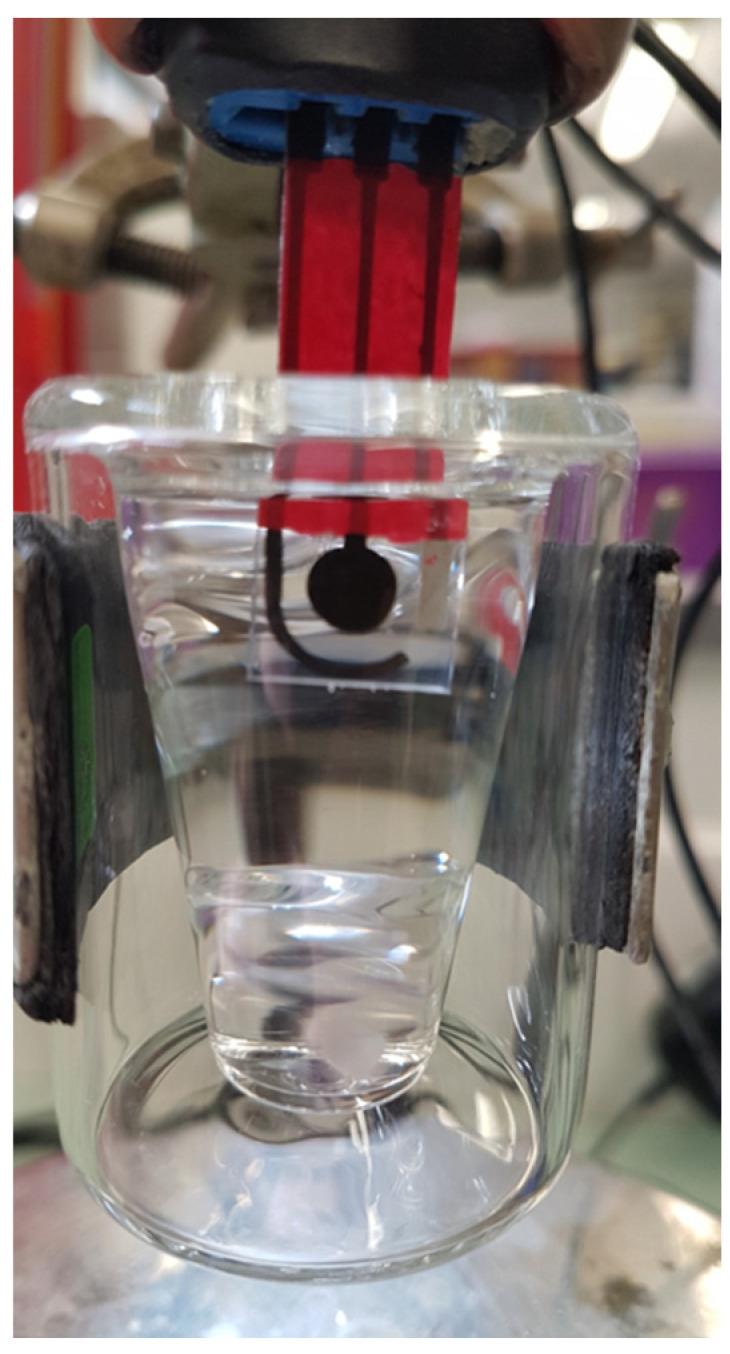
Schematic representation of the biosensor immersed in the thermostated cell.

Chronoamperometric measurements were carried out in a 10 mL thermostated cell using a PG581 Uniscan potentiostat (Uniscan Instruments, Buxton, UK). The biosensor was immersed in 10 mL of PBS buffer at pH 7 and a potential of 0.1 V versus Ag/AgCl was applied, corresponding to the oxidation potential of CoPC (Figure 2). Upon the addition of 100 µL of acetylthiocholine chloride at 1 mM, the oxidation current increased until reaching a plateau corresponding to the steady-state response. Such measurement was repeated four times to confirm the stability of the biosensor response. For inhibition experiments, 1 mL of biocide solution was added to 9 mL of PBS buffer. The biosensor was incubated for 10 min in this mixture, and its residual response was measured as described above. The cell was washed with PBS between measurements. The inhibition rate was then calculated using the equation (I_0_−I_(biocide)_)/I_0_, where I_0_ and I_(biocide)_ correspond to the intensity of the current measured in absence and in presence of biocide, respectively. Calibration curves were established using known concentrations of BAC and DDAC. To estimate the importance of matrix effect, inhibition experiments were carried out using biocides solutions prepared in deionized water or in tap water.

#### 2.2.3. Sample Pretreatment by Solid-Phase Extraction

An oasis HLB SPE cartridge was used due to its good extraction properties based on the hydrophobic interaction with the apolar parts of both biocides. The cartridge was preconditioned with 5 mL of methanol, followed by 4 mL of distilled water. DDAC and BAC biocides were solubilized in either distilled or tap water, and each biocide solution (500 mL) was directly injected into the cartridge at a flow rate of 4 mL/min. Elution was performed using 5 mL of methanol. Subsequently, methanol was evaporated using a rotary evaporator machine, and the biocides were finally diluted in 1 mL of water.

#### 2.2.4. Analyses of Samples Containing Organophosphate Pesticides

Various biocide concentrations and paraoxon (PO) at 10 µM were incubated for 10 min in a thermostated cell containing 10 mL of PBS 0.1 M pH 8 in the presence of 0.8 U/mL of PTE. This step allowed for the hydrolysis of PO. After this incubation time, an AChE- or BChE-based biosensor was immersed in the cell, and the activity of the enzyme was measured upon the addition of 1 mM ATCh. The inhibition percentage was then calculated as previously described in Section 2.2.2 by comparison with the sensor initial activity. Both PTE treatment and biosensor measurement were made at pH 8 in the same buffer to avoid multiple steps and excessive buffer consumption [16]. In parallel, control tests were carried out in the same conditions but in the absence of PO and PTE. For comparison purposes, PO inhibition in the absence of QA biocide before and after treatment with PTE was also measured using both enzyme sensors.

## 3. Results

### 3.1. Optimization of Operating Temperature

The effect of temperature on enzymes is well known and has been widely described [23]. Therefore, in this study, we performed amperometric measurements at different temperatures, notably at 24 °C (room temperature), 30 °C, and 40 °C, to determine the optimal temperature of each enzyme used.

**Figure 3:**
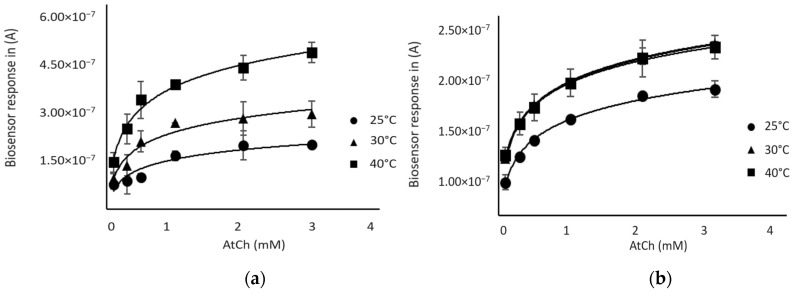
Response of the biosensor as a function of substrate (AtCh) concentration at different temperatures: (**a**) BChE-based biosensor (**b**) AChE-based biosensor.

As shown in Figure 3, the biosensor response increased with temperature using both enzymes; a plateau was observed at 30 °C for the AChE-based biosensor, while the BChE-sensor response continued to increase between 30 °C and 40 °C. However, a better reproducibility of sensor responses was observed for both sensors at 30 °C, so this temperature was chosen for further experiments.

### 3.2. Biosensor Detection of BAC and DDAC

#### 3.2.1. Calibration Curves

**Figure 4:**
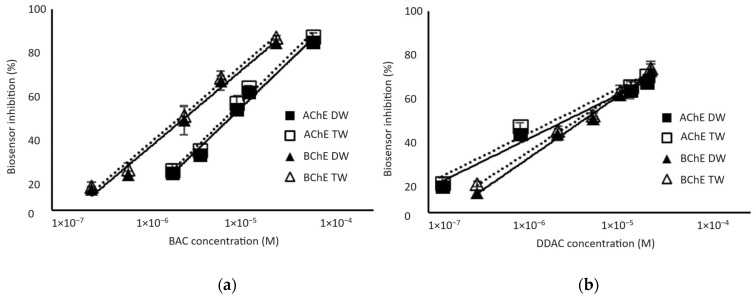
Inhibition effect of BAC (a) and DDAC (b) biocides on AChE- and BChE-based biosensors. Biocides were either prepared in distilled water (DW) or tap water (TW). Equations of the obtained curves are the following: (**a**) AChE-DW: y = 19.273ln(x) + 271.23 (R^2^ = 0.9864); AChE-TW: y = 19.625ln(x) + 277.62 (R^2^ = 0.9863); BChE-DW: y = 16.775ln(x) + 261.12 (R^2^ = 0.9883); BChE-TW: y = 16.952ln(x) + 265.6 (R^2^ = 0.9913). (**b**) AChE-DW: y = 9.7538ln(x) + 176.67, R^2^ = 0.9839); AChE-TW: y = 9.8656ln(x) + 181.03 (R^2^ = 0.9736); BChE-DW: y = 13.757ln(x) + 221.11 (R^2^ = 0.9935); BChE-TW: y = 12.983ln(x) + 213.91 (R^2^ = 0.9920).

Biosensors calibrations were performed for standard biocide concentrations diluted in either distilled or tap water to determine the presence or absence of matrix effects. As shown in Figure 4, the percentage of inhibition was not affected by the matrix when tap water was used instead of distilled water. The limits of detection (LODs) of the biosensor, calculated as the biocide concentration inducing a 10% decrease in the sensor response, were 1.3 µM and 0.32 µM for BAC biocide using AChE and BChE enzymes, respectively. The biosensors showed a better sensitivity to DDAC biocide, with LODs of 0.038 µM and 0.22 µM using AChE and BChE, respectively. It is interesting to notice that the AChE sensor showed a higher sensitivity to DDAC, while the BChE sensor was more sensitive to BAC. It is important to stress here that these concentrations correspond to the final concentration in the analytical cell after a 10-fold dilution of the sample.

Although the detection limits of the developed sensors are in micromolar range, the sensitivity of these devices was not compatible with the limit of 0.028 µM imposed by the European regulation. For this reason, a pre-concentration step was mandatory before biosensor analysis.

#### 3.2.2. Sample Pre-Concentration and Biosensor Analysis

500 mL of tap water spiked with known concentrations of biocide were passed through an HLB SPE cartridge, eluted with 5 mL of methanol, evaporated, and finally collected in 1 mL of tap water. The resulting concentrated extracts were then analyzed using the BChE- and AChE-based biosensors, and the obtained inhibition percentage allowed for the calculation of each biocide concentration based on the corresponding calibration curve equation. The measured concentration was then compared to the theoretical concentration, allowing for the calculation of recovery rates for each biocide tested. As shown in Table 1, the BChE sensor was suitable for evaluating the efficiency of BAC preconcentration in a wide concentration range, showing recovery rates between 78% and 109%. Similarly, DDAC was determined using both biosensors with recovery rates ranging between 74.8% and 99.3% (Table 2). These results confirm the efficiency of the HLB SPE concentration of BAC and DDAC compounds in a wide concentration range, suitable with targeted values.

Taking into account that the biocide samples were 500-fold concentrated through SPE extraction, and that the biosensor measurement involves a 10-fold dilution of the injected solution, the actual range of concentrations measurable by the developed method were calculated (Table 3). These results show that the wider range of biocide concentration is detected using the BChE sensor for BAC and using the AChE sensor for DDAC. Using these biosensors, the limits of quantification obtained for BAC and DDAC are, respectively, 5.3 nM and 2.7 nM. Keeping in mind that the maximum concentration tolerated in food industry wash waters is 28 nM, the developed biosensors were sensitive enough to detect DDAC and BAC biocides, regardless of the enzyme used.

### 3.3. Biosensor Stability

**Figure 5:**
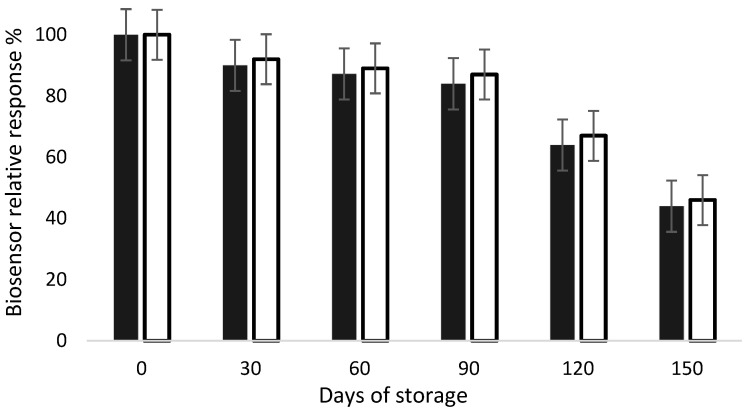
Relative response of AChE (dark bars) and BChE (white bars) biosensors during storage at 4 °C. The biosensor response was tested in presence of acetylthiocholine at 1 mM, as described in inhibition experiments.

Biosensors are devices susceptible to aging due to the presence of biological receptors, which are well known for having a short shelf life. This event is characterized by the decrease in the sensor’s signal over time. Therefore, this feature is of major importance for the success of this device for commercial use. Stability characteristics related to shelf life are often poorly investigated or reported in the literature [24]. Hence, in this work, stability tests were performed using the developed biosensors. AChE- and BChE-modified electrodes were fabricated and stored for a 5-month period at 4 °C, and their response to 1 mM acetylthiocholine was measured every 30 days (*n* = 3 electrodes). As presented in Figure 5, the biosensors response remained stable for 3 months, regardless of the enzyme used. A significative decrease in the signal was noticed after 4 months storage, resulting in a 50% loss after 5 months. These results show that both AChE- and BChE-based biosensors can be stored at 4 °C for at least 3 months without a significative loss of activity.

### 3.4. Analyses of Samples Containing Organophosphate Pesticides

#### 3.4.1. Evaluation of PTE Activity in the Presence of BAC and DDAC

It is well known that certain insecticides, in particular organophosphates, are powerful inhibitors of cholinesterases. Their presence in samples may result in a rapid and irreversible inhibition of the response of cholinesterase sensors [25]. Even though the wash waters used in food industry are normally free of pesticides, we thought about addressing this possibility by using an additional enzyme named phosphotriesterase (PTE) which is able to hydrolyze organophosphate esters, including organophosphorus insecticides. For this purpose, paraoxon (PO) insecticide was selected as a model substrate of PTE because its hydrolysis leads to the production of paranitrophenol (PNP), which can be easily detected by spectrophotometry due its yellow color. The effect of BAC and DDAC biocides on PTE activity was first studied by carrying out kinetics at 405 nm. As described in Table 4, the results clearly showed that BAC and DDAC biocides have no effect on the hydrolase activity of PTE, even at concentrations as high as 1000 µM. Based on these observations, PTE appears as an appropriate tool for eliminating organophosphorus insecticides without impairing the sensitivity of the ChE sensor to quaternary ammonium biocides.

#### 3.4.2. Effect of PTE Treatment on the Biosensor Detection of BAC and DDAC in Presence of PO

**Figure 6:**
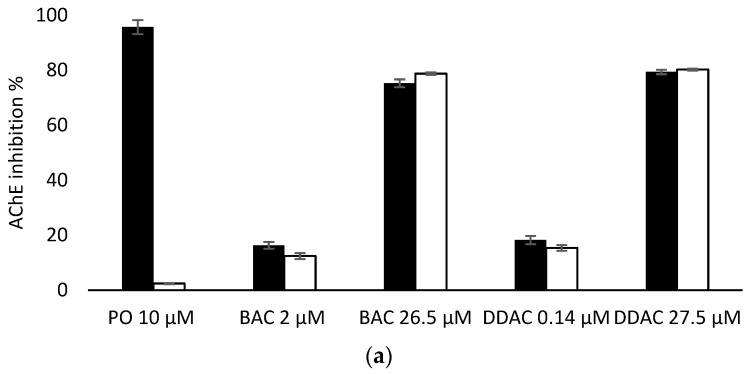

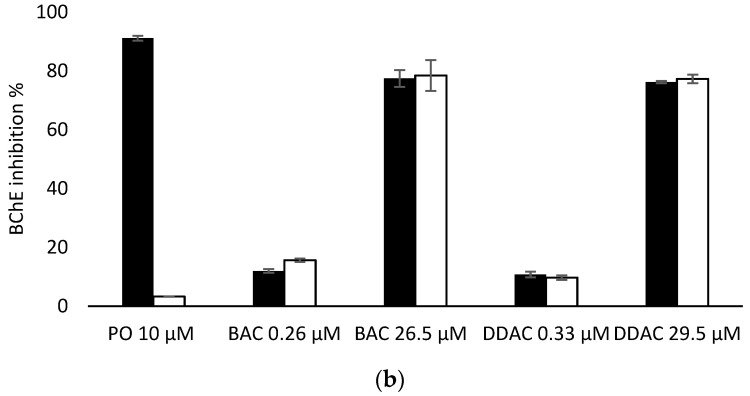
Comparison of AChE (**a**) and BChE (**b**) sensor inhibition by BAC and DDAC at different concentrations, in absence (dark bars) or presence (white bars) of PTE at 0.8 U/mL and PO insecticide at 10 µM.

Having demonstrated that PTE has no effect on the inhibition of the two biosensors by BAC and DDAC biocides, biosensors measurements were carried out in presence of PO at 10 µM to evaluate the efficiency of PTE treatment and its possible use as a tool for eliminating potential interferences due to organophosphorus pesticides. In these assays, a PO concentration of 10 µM was chosen as it induces a total inhibition of both sensors (Figure 6). It was demonstrated that a 10 min treatment of a 10 µM PO solution with PTE at 0.8 U/mL was sufficient to completely eliminate the effect of this pesticide, regardless of the enzyme used (Figure 6). BAC and DDAC solutions were prepared at concentrations close to the lower and upper values of the quantification range for each sensor, and the sensors’ inhibitions in the absence and presence of PTE and PO at 10 µM were compared. As shown in Figure 6a,b, the results showed percentages of inhibition very similar to those obtained for the biocides alone, demonstrating that the PTE treatment allows for the obtainment of reliable results, even if potent cholinesterase inhibitors like PO are incidentally present in the sample. As expected, the presence of PTE in the reaction medium effectively prevents the action of such inhibitors which could jeopardize the reliability of the sensor.

## 4. Conclusions

A biosensor should acquire certain characteristics to be used in the real world. In this work, we developed cholinesterase-based biosensors capable of detecting BAC and DDAC biocides in tap water at very low concentrations. Calibrations experiments carried out either with distilled water or tap water samples showed the absence of matrix effects, regardless of the enzyme and biocide used. A simple SPE preconcentration step allowed for the attainment of limits of quantification in the nanomolar range compatible with regulations applied to food industry wash waters. The cholinesterase biosensors were also used to evaluate the efficiency of the preconcentration step, and satisfying recovery rates were obtained, ranging from 78% to 100%. In addition, the developed biosensors exhibited good storage stability for over three months. Additional experiments in the presence of phosphotriesterase showed that this enzyme can be efficiently used to remove organophosphate compounds and therefore eliminate potential false-positives or interferences due to these potent inhibitors. The results presented in this paper show that cholinesterase-based biosensors are promising tools for the simple and sensitive detection of quaternary ammonium biocides in food industry wash waters. Future works will focus on the possibility of using artificial neural networks for analyzing complex biocide mixtures using biosensors arrays, as already described in our group for discriminating mixtures of organophosphate insecticides [26].

## Figures and Tables

**Table 1:** Recovery rates obtained for BAC after SPE preconcentration, determined either using AChE- or BChE-based biosensors (concentrations are those of biosensor measurement cell).

AChE Biosensor y = 19.625ln(x) + 277.62			BChE Biosensor y = 16.952ln(x) + 265.6		
(BAC)_theoretical_ µM	(BAC)_measured_ µM	Recovery (%)	(BAC)_theoretical_ µM	(BAC)_measured_ µM	Recovery (%)
0.27	-	-	0.27	0.29	109
0.67	-	-	0.67	0.61	91.7
2.65	2.30	86.8	2.65	2.18	82.2
26.5	22.5	86.5	26.5	20.3	78.1

**Table 2:** Recovery rates obtained for DDAC after SPE preconcentration, determined either using AChE- or BChE-based biosensors (concentrations are those of biosensor measurement cell).

AChE Biosensor y = 19.625ln(x) + 277.62			BChE Biosensor y = 16.952ln(x) + 265.6		
(DDAC)_theoretical_ µM	(DDAC)_measured_ µM	Recovery (%)	(DDAC)_theoretical_ µM	(DDAC)_measured_ µM	Recovery (%)
0.14	0.11	81.5	0.14	-	-
0.34	0.25	75.8	0.34	0.27	74.8
0.75	0.62	80.0	0.75	0.74	99.3
2.70	2.2	81.5	2.70	2.60	96.3
29.7	23.7	89.4	29.7	27.0	90.9

**Table 3:** Range of actual concentrations of BAC and DDAC contained in tap water determined after SPE concentration and biosensor analysis using either AChE- or BChE-based sensors.

	BAC	DDAC
**AChE sensor**	0.053–0.53 µM	0.0027–0.59 µM
**BChE sensor**	0.0053–0.53 µM	0.0067–0.59 µM

**Table 4:** Effect of increasing concentrations of BAC and DDAC quaternary ammoniums on PTE activity using paraoxon as substrate (*n* = 3 assays).

Biocide Concentration (µM)	PTE Relative Activity %	
	BAC	DDAC
0	100 ± 7.81	100 ± 0.86
10	9 ± 4.10	102.05 ± 0.97
100	89.30 ± 6.81	102.81 ± 1.06
1000	86.90 ± 2.97	88.73 ± 2.24

## Data Availability

Data is contained within the article.
